# Injuries Associated with Recreational and Sport Angling: A Case Series of Mechanisms, Diagnosis, Treatment, and Rehabilitation

**DOI:** 10.3390/healthcare14182977

**Published:** 2026-09-11

**Authors:** Paweł Pędrasik, Bartosz Wilczyński, Monika Kontowt, Katarzyna Zorena

**Affiliations:** 1 Department of Immunobiology and Environmental Microbiology, Faculty of Health Sciences, Medical University of Gdańsk, 80-210 Gdańsk, Poland; 2 Seaside Medical Center Ltd., Medical Home, Kołobrzeska Street 46, 80-394 Gdańsk, Poland

**Keywords:** sport and recreational fishing, ocular trauma, musculoskeletal disorders, penetrating hand injury, rotator cuff tendinopathy, lateral epicondylitis

## Abstract

**Background:** Angling-related injuries include acute trauma and chronic overuse conditions, but detailed clinical descriptions of different injury mechanisms remain limited. This retrospective case series presents four clinically distinct injuries associated with recreational and sport angling. **Methods:** Four purposively selected patients were recruited between 6 November 2025 and 30 April 2026 through questionnaires, angling forums, events, and regional angling associations. All provided written informed consent and sufficient medical documentation. Clinical data were obtained from participant-provided medical documentation, imaging reports, and interviews, with follow-up varying according to the clinical course. **Results:** Cases included high-velocity open-globe ocular trauma, a penetrating palmar injury caused by a baiting needle, a penetrating finger injury caused by a large barbed double hook, and rotator-cuff tendinopathy with lateral epicondylitis in the context of high angling exposure. Management included surgery, antimicrobial treatment, rehabilitation, and load modification. Outcomes ranged from substantial visual recovery to persistent scar-related hand limitation and improvement in overuse symptoms. **Conclusions:** These cases illustrate projectile, penetrating, and repetitive-load injury mechanisms associated with angling and support clinical awareness, careful assessment of equipment-related trauma, and activity modification and rehabilitation in overuse conditions. Preventive measures discussed should be regarded as clinical considerations and hypotheses for further study rather than interventions of established effectiveness.

## 1. Background

Recreational angling and sport fishing are among the most widely practiced outdoor activities worldwide and are increasingly studied for their health effects [1,2,3,4,5,6,7]. These activities may provide psychological, social, and physical benefits [8,9,10,11,12,13,14].

However, angling is also associated with a broad spectrum of injuries, ranging from acute trauma caused by hooks, lures, and other equipment to chronic musculoskeletal disorders related to repetitive casting and prolonged upper-limb loading [2,15,16,17,18,19,20].

Ocular injuries may result from high-velocity hooks, lures, sinkers, or tensioned fishing lines and can require urgent ophthalmologic and surgical management [16,21,22,23,24,25,26,27].

Repetitive casting and prolonged rod handling have also been associated with upper-extremity symptoms involving the shoulder, elbow, wrist, and hand [6,28,29,30]. In addition, anglers frequently traverse uneven terrain while carrying equipment; prolonged standing, cold exposure, wading in resistant water environments, and unstable substrates further increase biomechanical strain and fatigue [31,32,33,34].

Penetrating injuries most commonly involve the hands and fingers and may be complicated by deep foreign-body retention, contamination, or involvement of tendons, nerves, vessels, joints, or bone [15,35]. Removal often requires specialized techniques, including advance-and-cut or modified clamp-and-retract approaches, and may be complicated by infection risk or involvement of tendon sheaths and neurovascular structures [36,37,38,39,40,41,42,43].

Although angling-related injuries have been described in case reports and emergency-department surveillance studies, the available literature generally focuses on individual injury types or mechanisms. Detailed clinical reports comparing acute projectile trauma, penetrating equipment-related injuries, and chronic repetitive-load disorders within a single case series remain limited. The present study therefore describes four clinically distinct cases and organizes them according to their predominant injury mechanism—projectile, penetrating, or repetitive-load injury—to facilitate comparison of their clinical presentation, diagnostic assessment, management, rehabilitation, and outcomes.

## 2. Methods

The cases included in this series were identified within a broader research initiative examining health outcomes and injuries associated with sport and recreational angling. Ethical approval for the broader research project was obtained from the Bioethics Committee of the Medical University of Gdańsk (Approval No. KB/155/2024, dated 5 July 2024). The approved study procedures included medical history taking, clinical examination, and interviews with participants. Depending on the nature and scope of the assessment, clinical and functional examinations could be performed by either a physician or a physiotherapist. Review of prior medical documentation formed part of the clinical assessment and was based exclusively on documentation voluntarily provided by the participants themselves; the authors did not obtain medical records directly from healthcare institutions or institutional medical-record systems.

Follow-up interviews conducted for the purposes of the present case series were undertaken within the interview-based component of the approved study. The approved protocol also included questionnaire-based recruitment, clinical and functional assessment, and diagnostic evaluation of angling-related injuries.

Written informed consent for participation in the study and for the use of participant-provided medical documentation was obtained from all patients in accordance with the principles of the Declaration of Helsinki. Separate, explicit written consent for publication of potentially identifiable clinical photographs was also obtained from all participants.

This case series was prepared in accordance with the CARE (CAse REport) guidelines for clinical case reporting [44], and the completed CARE checklist is provided as a Supplementary File.

This study represents a descriptive, retrospective case series aimed at illustrating clinically distinct mechanisms of angling-related injuries. Recruitment was conducted from 6 November 2025 to 30 April 2026 through a multimodal strategy that included questionnaire-based recruitment, dedicated online angling forums, on-site recruitment during angling events, and direct in-person contact through the Gdańsk and Bydgoszcz Districts of the Polish Angling Association. Recruitment and data collection were completed on 30 April 2026, before submission of the manuscript. 

The questionnaire formed part of the broader research project and included 169 respondents. Of these, 124 reported a clinically relevant angling-related injury. Among the 124 respondents reporting a clinically relevant angling-related injury, 11 had consented to be contacted regarding potential further participation in the study. These individuals were subsequently contacted and invited to provide available medical documentation for clinical verification and potential inclusion in the case series. Seven declined further participation, and two were excluded because the available documentation was insufficient for reliable clinical verification and detailed case reconstruction. Ultimately, two questionnaire respondents were included in the present case series.

The recruitment source differed between cases: Case 1 was identified during an angling event in Gdańsk on 15 November 2025, Case 2 through direct in-person recruitment via the Polish Angling Association on 4 January 2026, and Cases 3 and 4 through the study questionnaire. Because the present study was retrospective, the dates of the original clinical events preceded study recruitment.

Case 1 concerned an injury and initial treatment on 12 May 2024, before ethical approval was obtained. The participant was subsequently identified and approached for recruitment on 15 November 2025, when written informed consent was obtained. The participant was formally enrolled in the study on 17 November 2025. An investigator-conducted interview was performed on 20 November 2025. 

Case 2 concerned an injury and initial treatment on 15 June 2023. The participant was identified and approached for recruitment on 4 January 2026 and provided written informed consent on the same day. He voluntarily supplied the relevant prior medical documentation on 5 January 2026 and was formally enrolled in the study on 6 January 2026. A retrospective follow-up interview was conducted on 15 April 2026. 

Case 3 concerned an injury and initial treatment on 10 August 2024. The participant was subsequently identified through the study questionnaire and recruited on 9 April 2026. Written informed consent was obtained on 10 April 2026. The relevant prior medical documentation was voluntarily provided on 11 April 2026. The participant was formally enrolled in the study on 13 April 2026. A retrospective follow-up interview was conducted on 15 April 2026.

Case 4 concerned an initial clinical presentation on 5 May 2020. The participant was identified and recruited on 20 January 2026. Written informed consent was obtained on 10 February 2026, and the relevant prior medical documentation was voluntarily provided on 11 February 2026. The participant was formally enrolled in the study on 14 April 2026. A retrospective follow-up interview was conducted on 15 April 2026.

The original clinical management of all four cases occurred independently of the present research project. Their subsequent inclusion in the case series was retrospective and took place only after ethical approval had been obtained. In all cases, the medical documentation was provided directly by the participants and was not obtained from healthcare institutions or institutional medical-record systems. Participant-provided medical documentation was used to verify and reconstruct the prior clinical course.

The four cases were not consecutive but were purposively selected as illustrative examples representing clinically distinct injury mechanisms and clinical scenarios rather than their epidemiological frequency. Although participant flow from the questionnaire arm could be reconstructed, the exact total number of potentially eligible individuals screened across the other recruitment channels, together with individual reasons for non-inclusion, could not be reliably reconstructed because case identification was multimodal and retrospective.

Patient perspectives were collected retrospectively during follow-up interviews and are presented as author-prepared summaries rather than verbatim quotations.

### 2.1. Data Collection

Clinical information for each case was obtained through patient interviews and review of the available medical documentation. Information obtained from patient interviews was used primarily to reconstruct the circumstances and mechanism of injury, environmental conditions, angling technique, equipment involved, and relevant exposure history. Clinical findings, diagnoses, treatment details, surgical procedures, pharmacological management, and follow-up information were extracted from the available participant-provided medical documentation.

The available medical documentation and written diagnostic imaging reports were reviewed by the first author, a qualified paramedic and physiotherapist with extensive practical experience in recreational and sport angling, and by a second clinician, an experienced surgeon with many years of clinical practice. This combination of clinical expertise and angling-specific knowledge supported the assessment of the documented clinical course, injury mechanisms, equipment characteristics, and circumstances of the incidents. Original diagnostic images were not available for direct review in all cases; therefore, imaging findings were based on the corresponding written radiological or ultrasonographic reports contained in the source documentation.

Data collection focused on the mechanism and circumstances of injury, including environmental conditions and the type of angling equipment involved, as well as findings from the physical examination relevant to ocular, musculoskeletal, or hand trauma. The clinical course was documented in detail, including emergency management, specialist consultations, surgical procedures where applicable, pharmacological treatment, rehabilitation interventions, and follow-up outcomes. Follow-up information was used to assess recovery, residual functional limitations, complications, and return to angling activity. Patient-reported information was distinguished from information documented in the participant-provided medical documentation wherever possible.

**Figure 1:**
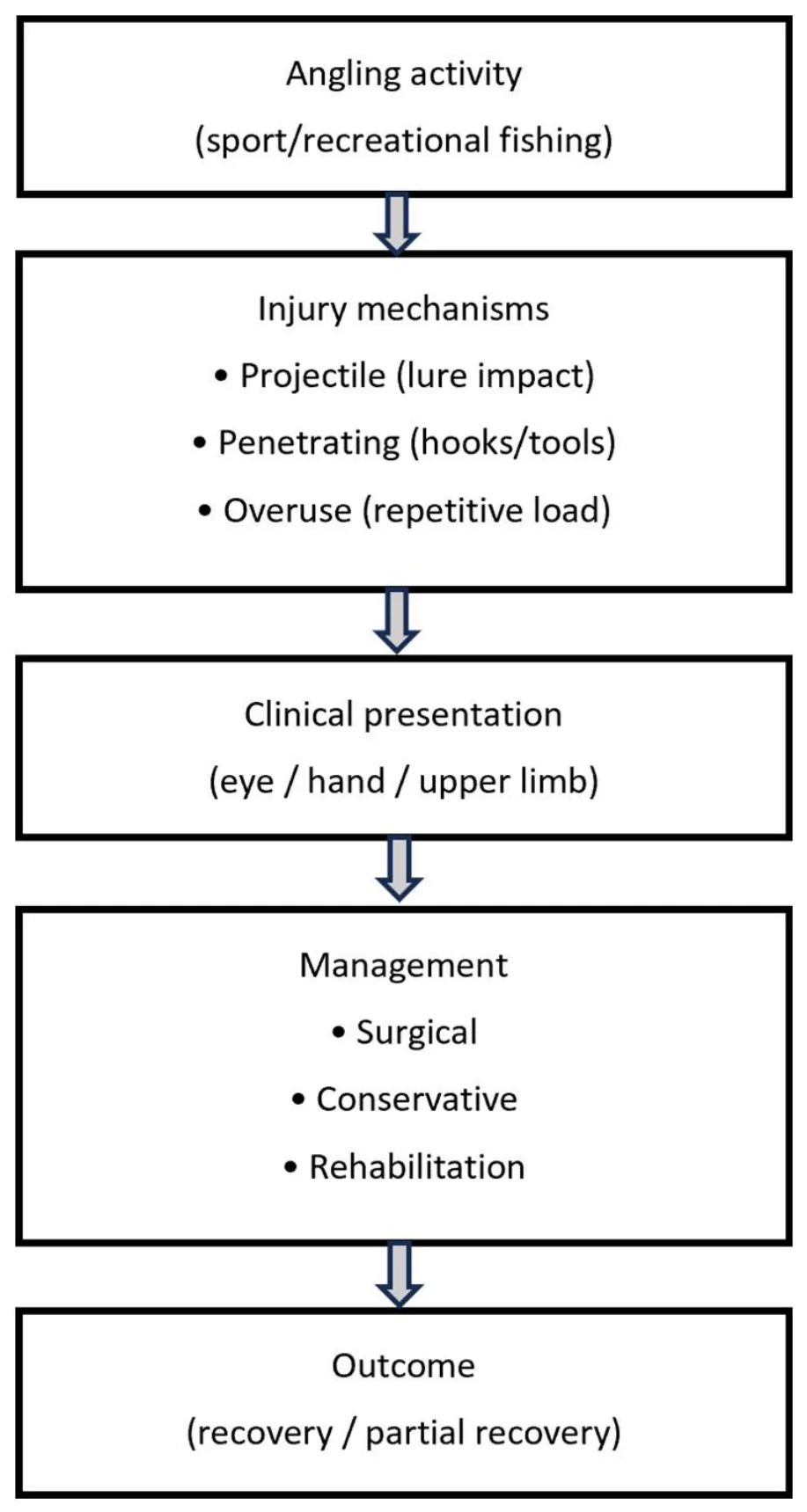
Author-developed descriptive schematic of the predominant angling-related injury mechanisms represented in the present case series.

Figure 1 was developed by the authors as a descriptive schematic based on the predominant injury mechanisms represented by the four included cases. It was not used as a validated classification system or as a formal eligibility criterion. Rather, it served as an organizational framework to group the cases into three broad mechanism categories—projectile, penetrating, and repetitive-load injuries—and to facilitate comparison of their clinical presentation, management, and outcomes.

### 2.2. Inclusion Criteria

Cases were eligible for inclusion when the injury occurred directly during recreational or sport angling activities, such as casting, lure retrieval, equipment handling, landing fish, or bait preparation. Inclusion additionally required sufficient clinical documentation to allow accurate description of presentation, diagnostic evaluation, management, and outcome. Written informed consent for scientific publication of the clinical information and case details was obtained from all patients prior to inclusion. Separate explicit written consent was obtained for publication of potentially identifiable clinical photographs.

### 2.3. Exclusion Criteria

Cases were excluded when the injury was unrelated to angling activity, including incidents occurring during transport or other non-fishing contexts. Cases were also excluded if available clinical documentation was insufficient to ensure reliable reconstruction of the injury mechanism or clinical course. Injuries sustained exclusively during professional or commercial fishing activities were not considered, as the present series focused on recreational and sport angling. Finally, minor superficial injuries that did not require medical evaluation or therapeutic intervention were excluded in order to maintain clinical relevance.

## 3. Case Presentations

### 3.1. Case 1: High-Velocity Ocular Trauma Caused by Spinning Lure

A 34-year-old male recreational angler presented to the emergency department on 12 May 2024 with acute ocular trauma sustained while spin fishing in a river. The patient had no relevant ophthalmologic history. He had been using protective eyewear while fishing but had briefly removed it because of lens fogging shortly before the injury occurred. When retrieving the lure, it became snagged on a submerged obstacle. As the patient attempted to release the tackle by applying tension to the fishing line, the lure suddenly became dislodged and recoiled toward his right eye at high speed. Immediately after impact, he experienced periocular pain, photophobia, excessive tearing, and a sudden reduction in central vision.

On arrival at the emergency department, marked eyelid edema, subconjunctival hemorrhage, and a partial-thickness eyelid laceration caused by the treble hook were observed. Slit-lamp examination demonstrated a 3-mm full-thickness corneal laceration, a shallow anterior chamber, a positive Seidel test, and traumatic hyphema occupying approximately 25% of the anterior chamber. Visual acuity was limited to hand-motion vision (HM). Intraocular pressure was not measured at initial presentation because of the confirmed open-globe injury. Non-contrast orbital computed tomography demonstrated a metallic component of the hook extending through the corneal wound into the anterior chamber, consistent with an open-globe injury. No CT evidence of posterior-segment involvement was identified, and the lens appeared intact. Following primary globe repair, dilated fundus examination revealed no retinal, choroidal, or other posterior-segment abnormalities.

The open-globe injury was classified as a Zone I penetrating injury, as the full-thickness wound was confined to the cornea without documented scleral involvement. A formal Ocular Trauma Score (OTS) was not recorded at the initial presentation. Although several OTS components could be reconstructed retrospectively, the presence or absence of a relative afferent pupillary defect (RAPD) was not documented, precluding reliable calculation of the complete OTS.

Urgent ophthalmic surgery was performed, including removal of the embedded treble hook, primary closure of the corneal laceration with sutures, reconstruction of the anterior chamber, and anterior chamber washout with evacuation of the hyphema. Postoperatively, the patient received intravenous vancomycin 1 g every 12 h plus ceftazidime 2 g every 8 h for 24 h, followed by oral moxifloxacin 400 mg once daily for seven days. This regimen reflected an institution-specific, individualized clinical decision by the treating ophthalmic team for a contaminated penetrating open-globe injury and should not be interpreted as a general treatment recommendation. Topical moxifloxacin 0.5% was administered as one drop three times daily for seven days. Dexamethasone 0.1% eye drops were administered four times daily and gradually tapered according to the inflammatory response. Cyclopentolate 1%, one drop three times daily, was prescribed for several days to relieve ciliary spasm, pain, and photophobia. The patient was closely monitored for changes in intraocular pressure and signs of infectious or retinal complications. He remained hospitalized for 24 h postoperatively for close observation and was discharged after clinical stabilization.

Postoperative follow-up was performed on the first day after surgery and subsequently at approximately one, two, and six weeks, followed by assessments at three and six months. On the first postoperative day (13 May 2024), intraocular pressure was 14 mmHg. At approximately one week (20 May 2024), BCVA improved to 0.4 and IOP was 15 mmHg. BCVA remained stable at 0.4 during the early follow-up period, with IOP values of 15 mmHg at approximately two weeks and 14 mmHg at approximately six weeks. At the three-month follow-up, BCVA improved further to 0.5, with an IOP of 14 mmHg. At six months, BCVA reached 0.7 and IOP was 16 mmHg. Mild irregular astigmatism persisted, but no infectious complications, persistent hyphema, or posterior-segment pathology were identified.

The injury mechanism and clinical presentation were consistent with previously reported fishing-related projectile and penetrating ocular injuries involving hooks, lures, and tensioned fishing lines [21,22,35,40,45,46]. The injury mechanism highlights the potential importance of impact-resistant protective eyewear during lure casting and snag release [16].

### 3.2. Case 2: Penetrating Palmar Hand Injury Caused by a Fishing Baiting Needle During a Feeder Competition 

A 39-year-old right-handed male competitive angler presented to the emergency department on 15 June 2023 with a penetrating injury of the right hand sustained during a national feeder fishing competition. The patient had no prior history of hand injuries or musculoskeletal disorders. The injury occurred at the fishing station while the patient was reaching for accessories. His hand inadvertently pressed against a long stainless-steel baiting needle used for threading boilies, the handle of which had detached earlier, leaving the metallic shaft partially concealed among other equipment.

Initially, the patient experienced only mild discomfort and continued fishing. Several minutes later, during a forceful hook-set, he developed sudden severe pain localized to the palmar region of the hand, prompting immediate cessation of angling and transport to the emergency department.

Physical examination of the right hand demonstrated localized swelling, tenderness on palpation over the palmar region, and reduced grip strength secondary to pain. Radiographic imaging of the hand revealed a linear metallic foreign body, approximately 7 cm in length, located deep within the palmar soft tissues, without evidence of fracture or joint involvement.

The baiting needle entered the right hand through the palmar aspect of the fourth interdigital web space, between the ring and little fingers, and extended proximally through the palmar soft tissues toward the wrist, approximately parallel to the metacarpal shafts. No tendon, major neurovascular, or joint injury was identified during surgical exploration. This trajectory was confirmed on plain radiography and intraoperative fluoroscopy (C-arm). Surgical extraction of the retained baiting needle was performed on 15 June 2023 under regional anesthesia using intraoperative fluoroscopic guidance (C-arm) to ensure accurate localization and atraumatic removal of the foreign body. 

On postoperative day 1, the patient developed a fever of 39 °C, leukocytosis (WBC 16.2 × 10^9^/L), markedly elevated C-reactive protein (CRP 135 mg/L), and local inflammatory signs, raising concern for an evolving infectious process following a deeply penetrating and contaminated foreign-body injury. Wound culture was obtained at the time empirical intravenous antimicrobial therapy was initiated. Given the clinical and laboratory evidence of infection and the potential for polymicrobial contamination associated with freshwater exposure, including Gram-negative organisms, empirical intravenous amoxicillin–clavulanate was initiated at 1.2 g every 8 h before microbiological results were available. The wound culture subsequently yielded methicillin-susceptible *Staphylococcus aureus* (MSSA), which was susceptible to amoxicillin–clavulanate, supporting continuation of the same antimicrobial regimen on a culture-directed basis. Intravenous therapy was continued for five days and was then transitioned to oral amoxicillin–clavulanate 875/125 mg every 12 h for an additional five days. No re-exploration, drainage, or additional surgical debridement was required. A tetanus toxoid booster was administered. The six-day hospitalization from 15 to 20 June 2023 reflected the need for surgical foreign-body removal, intravenous antimicrobial therapy, postoperative observation, and monitoring for infectious and neurovascular complications.

Functional recovery progressed gradually, allowing the patient to return to normal daily activities. At the three-month follow-up in September 2023, the patient began a structured 8-week rehabilitation program, completed in November 2023, focused on restoration of finger range of motion, tendon gliding, scar mobilization, progressive grip-strengthening exercises, and recovery of fine motor function. At the beginning of rehabilitation, active finger flexion and grip strength of the affected right hand were limited. Grip strength was measured using a Jamar hand-grip dynamometer (Asp Global, LLC, Austell, GA, USA) with the patient seated, the shoulder adducted and neutrally rotated, the elbow flexed to 90°, and the forearm in a neutral position. Three trials were performed for each hand, and the mean of the three measurements was recorded. Grip strength was 25 kg, compared with 43 kg on the contralateral side. The fingertip-to-distal palmar crease distance during maximal active flexion was 3.0 cm, the active extension deficit was 20°, and pain during forceful gripping was rated 6/10 on the NRS. After completion of physiotherapy in November 2023, grip strength of the right hand increased to 37 kg, the fingertip-to-distal palmar crease distance decreased to 0.5 cm, the extension deficit decreased to 5°, and pain during gripping decreased to 2/10. Despite improvement in finger mobility and grip function, a mild residual limitation associated with palmar scar contracture persisted. In June 2024, approximately 12 months after the injury, scar revision surgery was performed to improve tissue mobility and hand function. Following the procedure, the patient underwent a further 8-week course of structured hand rehabilitation from July to September 2024, including scar mobilization, active and passive range-of-motion exercises, tendon-gliding exercises, and progressive grip-strengthening and functional training.

**Figure 2:**
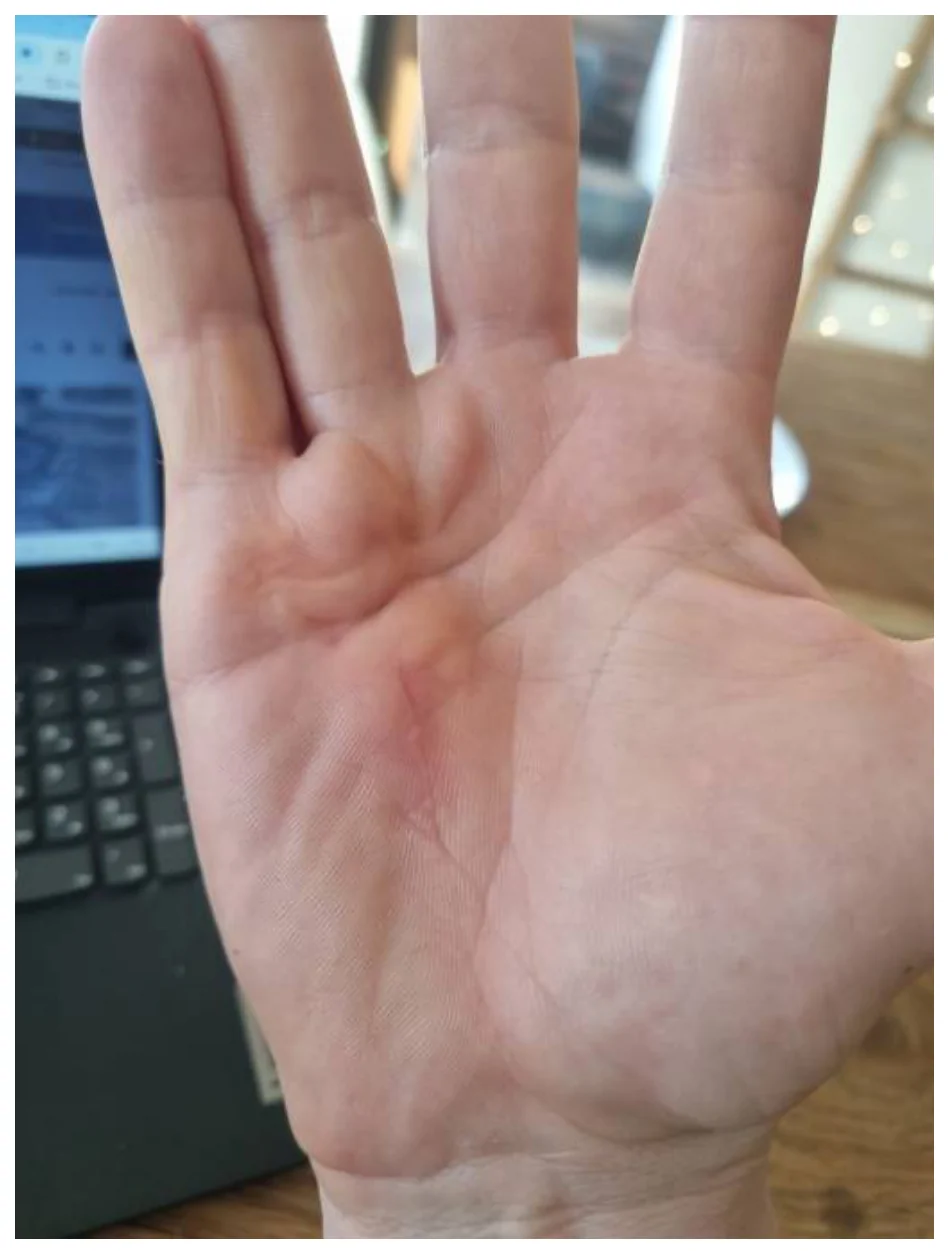
Clinical appearance of the injury site two years after needle removal. [Photograph by Paweł Pędrasik].

Despite further improvement in hand mobility and grip function, persistent post-traumatic palmar scar contracture continued to cause residual functional limitation. Full recovery had therefore not been achieved. At the clinical follow-up in July 2025, no further scar revision surgery had been performed, although hand function had further improved, residual palmar scar contracture and functional limitation persisted, and an additional corrective procedure was being considered (Figure 2).

At the investigator-conducted follow-up on 15 April 2026, the patient completed the QuickDASH questionnaire, yielding a score of 22.7/100. No earlier QuickDASH assessments were available, precluding longitudinal comparison using this validated patient-reported outcome measure. The total follow-up period from the injury on 15 June 2023 to the investigator-conducted follow-up on 15 April 2026 was 34 months.

The injury was consistent with a deep penetrating hand injury caused by auxiliary angling equipment, in which a detached baiting-needle handle left a concealed sharp metallic shaft capable of nail-like soft-tissue penetration. Previous reports emphasize contamination assessment, safe storage and regular inspection of angling tools, and prompt surgical evaluation when deep penetration is suspected [43,47].

### 3.3. Case 3: Penetrating Soft-Tissue Injury of the Finger Caused by a Large Catfish Double Hook

**Figure 3:**
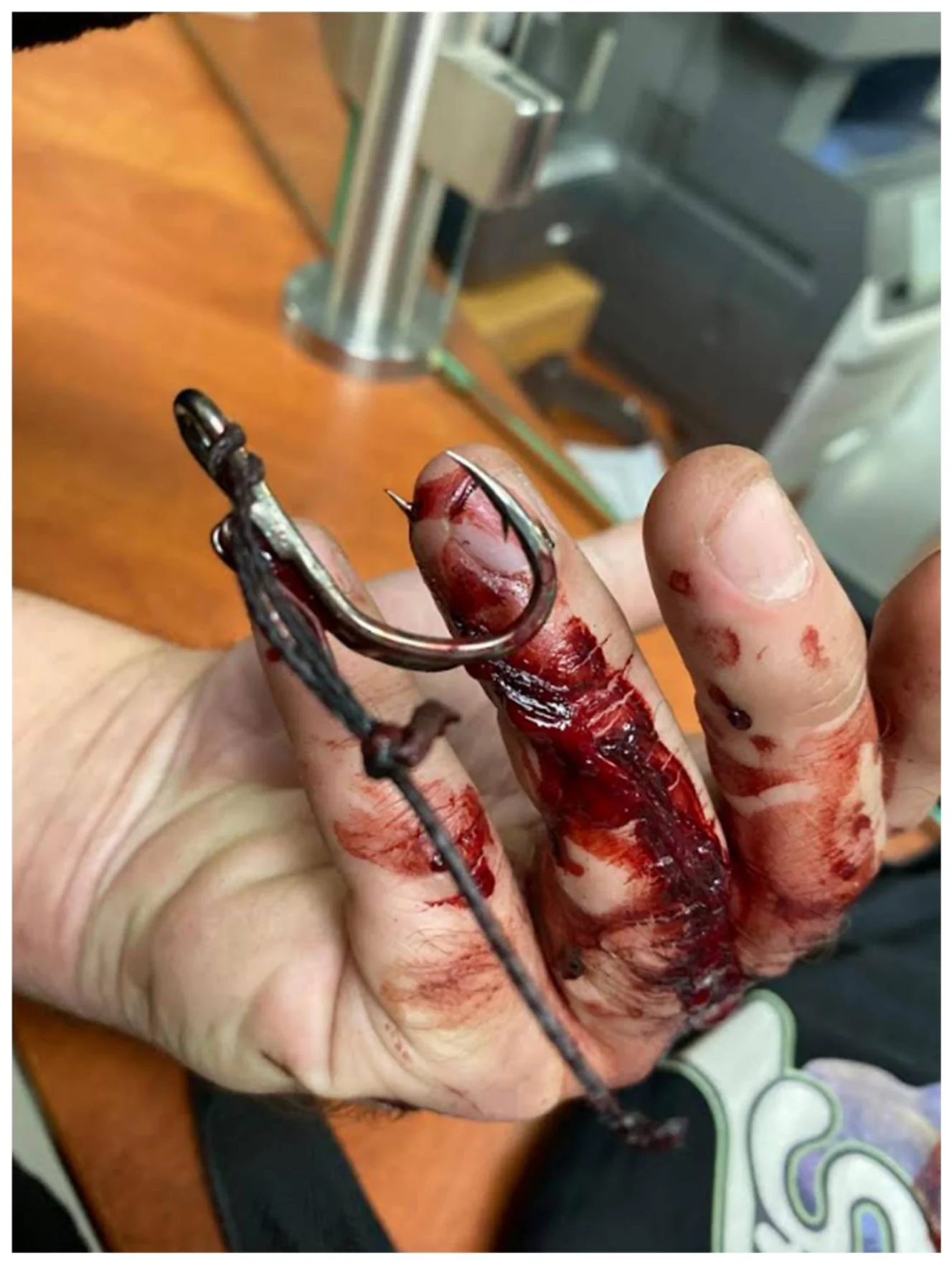
Transfixion of the left ring finger by a large barbed double hook, with clearly visible entry and exit points. [Photograph by Paweł Pędrasik].

A 40-year-old male competitive catfish angler presented to the emergency department on 10 August 2024 with a penetrating injury of the left ring finger sustained during a boat-based fishing competition. The patient had no prior history of hand injuries or relevant comorbidities. The injury occurred during a forceful hook-set while targeting large catfish, when the terminal tackle suddenly recoiled (“snap-back”), propelling a large double hook into the patient’s finger. The hook (size 10/0; approximately 9 cm × 6 cm; hardened alloy wire approximately 2.5 mm in diameter) penetrated the soft tissues, resulting in immediate severe pain and functional limitation of the affected finger (Figure 3).

Marked pain, localized swelling, and pain-limited range of motion were present. No active bleeding or signs of neurovascular compromise were observed. 

Because the foreign body was externally visible and its trajectory could be assessed clinically, additional radiographic imaging was not performed. Clinical examination showed preserved active finger motion despite pain-related restriction, without motor deficit and no clinical findings suggesting tendon, nerve, vascular, osseous, or joint involvement. However, occult osseous injury, articular penetration, or a retained foreign-body fragment could not be completely excluded in the absence of radiographic imaging. The decision not to obtain imaging was case-specific and should not be interpreted as a recommendation against radiography in similar injuries.

Initial attempts to divide the hook using standard emergency department instruments were unsuccessful because of the hardened alloy construction of the hook. Mechanical cutting with a grinding device was considered but rejected because of the risk of thermal injury to the surrounding tissues. The hook was removed under local anesthesia using a digital nerve block with 1% lidocaine. The source documentation did not record whether adrenaline was used. The surrounding skin was prepared with povidone–iodine before the procedure. A limited surgical approach with controlled extension of the wound tract was then performed, allowing safe extraction without excessive tissue manipulation or heat generation. Following extraction, the wound tract was thoroughly irrigated with sterile 0.9% saline; however, the exact irrigation volume was not documented in the source records. The hook was inspected and confirmed to be intact, indicating that no hook fragments had been retained. No gross tendon, joint, or neurovascular injury was identified during removal. The wound was subsequently closed with sutures.

Because the double hook penetrated the finger while the fish remained attached to the tackle, the wound was considered substantially contaminated with fish mucus, freshwater, and other aquatic organic material. Following hook removal, oral ciprofloxacin was prescribed at 500 mg every 12 h for 10 days because of the depth of penetration and concern regarding contamination with freshwater-associated Gram-negative organisms. No wound cultures or laboratory investigations were performed. Tetanus prophylaxis was administered. The antimicrobial regimen represented an individualized clinical decision based on the depth and degree of contamination and should not be interpreted as a routine recommendation for uncomplicated fishhook injuries.

**Figure 4:**
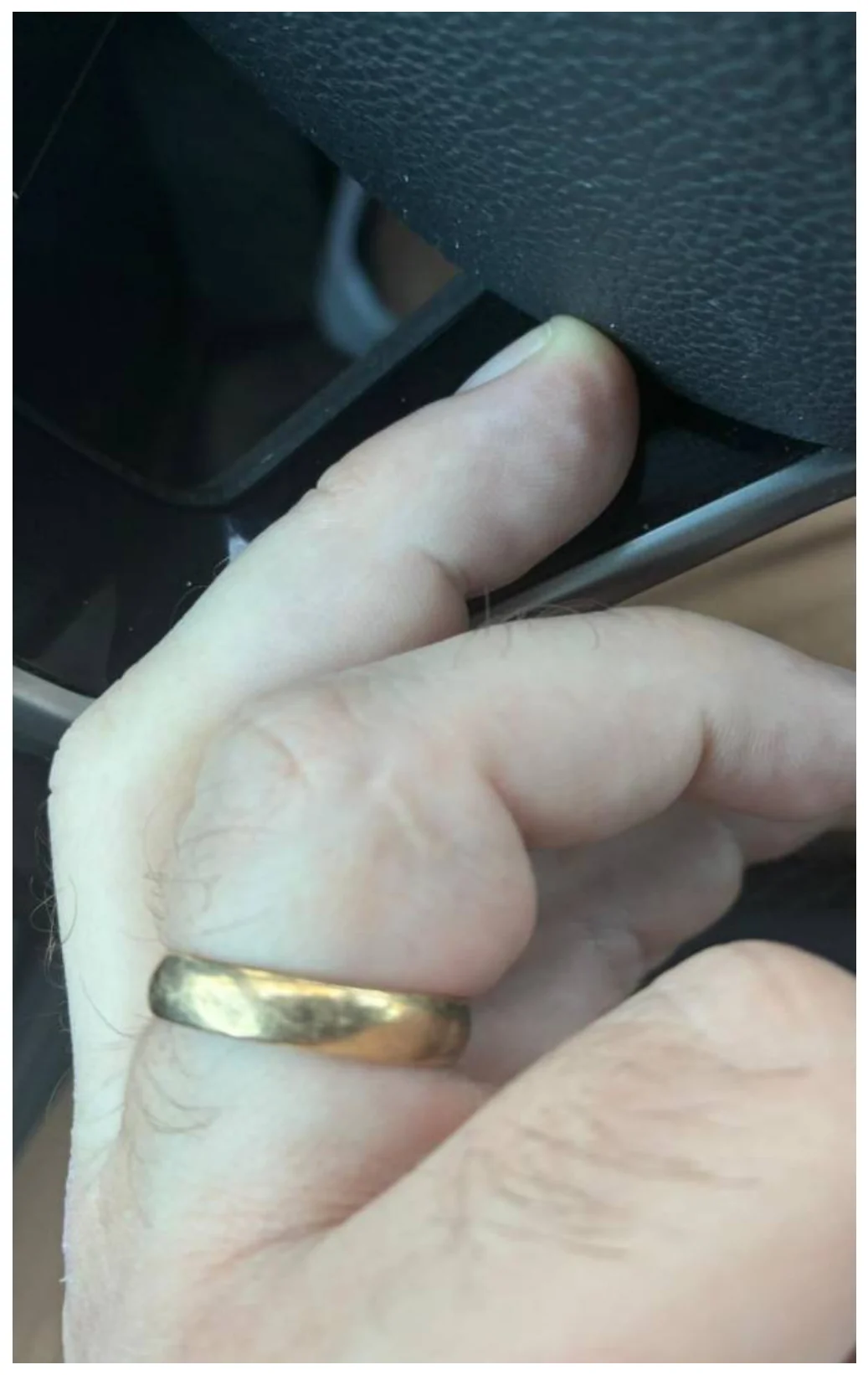
Clinical appearance of the surgical site 18 months postoperatively. [Photograph by Paweł Pędrasik].

The sutures were removed after 12 days following satisfactory wound healing. Wound healing progressed without complications. At the one-month follow-up assessment, preserved tendon function, intact sensory and motor function, and no vascular impairment were confirmed. The patient reported normal finger use without residual functional limitation and resumed angling activities within several weeks. A later clinical photograph obtained approximately 18 months after the procedure documented the appearance of the healed finger (Figure 4).

At the later investigator-conducted follow-up interview in April 2026, the patient reported no sensory disturbances, motor impairment, pain, or other problems related to the injured finger and described normal use without residual functional limitation, consistent with patient-reported complete recovery. No quantitative range-of-motion measurements, grip-strength testing, or validated patient-reported outcome measures were available at this follow-up.

### 3.4. Case 4: Chronic Rotator-Cuff Tendinopathy and Lateral Epicondylitis in a Year-Round Active Angler

On 5 May 2020, a 40-year-old right-handed male recreational angler presented with progressive right shoulder and lateral elbow pain that had been present for approximately 5–6 months of intensive, year-round fishing activity. The patient worked in a predominantly sedentary, computer-based occupation and reported no regular sports or strenuous upper-limb activities other than angling. Thus, no major additional sporting exposures were identified, although prolonged computer use was considered a potential non-angling occupational contributor to upper-limb symptoms. The patient had a history of two prior left knee arthroscopies but reported no other relevant comorbidities. He regularly engaged in year-round spinning using heavy lures and tackle, as well as summer angling targeting large catfish measuring up to approximately 2 m in length. He reported frequent fishing sessions with minimal recovery time, with questionnaire data indicating a self-reported retrospective estimate of approximately 200 fishing days per year. This value should therefore be interpreted as an approximate exposure estimate subject to recall error.

Symptoms gradually emerged in association with repetitive high-load casting, prolonged rod manipulation, and forceful fish retrieval, consistent with cumulative mechanical overload affecting the shoulder and elbow. The absence of adequate recovery periods appeared to contribute to progressive overload and symptom persistence. Clinical examination revealed pain localized to the lateral elbow and shoulder, discomfort during shoulder abduction and external rotation, and pain provoked by resisted wrist extension, consistent with lateral epicondyle involvement. Pain severity was rated as 7/10 for both the shoulder and elbow on an 11-point Numeric Rating Scale (NRS; 0 = no pain, 10 = worst imaginable pain). Active shoulder range of motion was mildly restricted by pain, particularly in abduction and external rotation. Range of motion was assessed clinically; no goniometric measurements were available. The documented examination therefore included provocative maneuvers involving shoulder abduction and external rotation and resisted wrist extension. Although resisted wrist extension overlaps conceptually with elements of the Cozen test, the exact standardized test position and technique were not documented. Specific named provocative tests for the shoulder and lateral elbow, including the Jobe (empty-can), Hawkins–Kennedy, Neer, Cozen, Mill, and Maudsley tests, were not explicitly recorded in the participant-provided medical documentation and therefore could not be reliably classified retrospectively as positive or negative. Functional limitations were evident during casting movements and sustained rod handling. Clinical examination was consistent with rotator-cuff tendinopathy. Ultrasonography demonstrated heterogeneous echogenicity and thickening of the supraspinatus tendon, findings compatible with tendinopathic changes, with no evidence of a full-thickness rotator-cuff tear.

Differential diagnoses considered for the shoulder symptoms included adhesive capsulitis and cervical referred pain or cervical radiculopathy. For the lateral elbow symptoms, radial tunnel syndrome and cervical radiculopathy were considered as alternative diagnoses. However, pain localized to the lateral epicondyle and reproduction of symptoms during resisted wrist extension supported the clinical diagnosis of lateral epicondylitis.

Conservative management was initiated with diclofenac 50 mg twice daily for 10 days, temporary reduction in provocative angling activity, and a 12-week structured physiotherapy program. Rehabilitation included pain-limited mobility exercises, scapular motor-control training, progressive strengthening of the rotator cuff and scapular stabilizers, isometric followed by concentric and eccentric loading of the wrist extensors, grip-strength and forearm-endurance exercises, and a graded return to casting with progressive increases in casting volume and tackle load. The rehabilitation program was supervised by a physiotherapist three times per week and was supplemented by a home-exercise program. Exercise load was progressed individually according to symptom response, movement quality, and exercise tolerance. Resistance was increased when exercises could be completed with correct technique, without sharp pain or sustained post-exercise symptom exacerbation, and with appropriate muscular fatigue.

The therapeutic response was initially limited while the patient continued intensive angling despite recommendations to reduce provocative activity. Subsequent clinical improvement coincided with a substantial reduction in angling exposure and completion of the rehabilitation program. At the 3-month follow-up, shoulder and elbow pain had decreased from 7/10 to 2/10 on the NRS. The patient reported substantial improvement in symptoms and upper-limb function and had resumed angling while adhering to load-management principles and a preventive exercise program. No post-treatment goniometric range-of-motion measurements, grip-strength measurements, or validated functional patient-reported outcome measures were available in the original source documentation. Despite the substantial improvement, intermittent recurrences of shoulder and lateral elbow symptoms were reported approximately 1–2 times per year, typically during periods of increased angling intensity or insufficient recovery.

The overall follow-up period from the initial presentation on 5 May 2020 to the investigator-conducted retrospective follow-up interview on 15 April 2026 was approximately 6 years. At this follow-up, the Shoulder Pain and Disability Index (SPADI) score was 10.8/100, while the Patient-Rated Tennis Elbow Evaluation (PRTEE) score was 0/100, indicating no current elbow-related pain or functional limitation. No earlier SPADI or PRTEE assessments were available for longitudinal comparison. A concise clinical timeline, demographic characteristics, and summary of all four cases are presented in Table 1, Table 2 and Table 3.

## 4. Patient Perspective

The patient with ocular trauma reported significant concern regarding potential permanent vision loss at the time of injury. He emphasized that he had not anticipated that such a seemingly minor circumstance could result in a severe injury requiring urgent surgical intervention. Although visual acuity subsequently improved substantially, the experience increased his awareness of the potential severity of angling-related ocular injuries and the importance of continuous use of protective eyewear.

The patient with the penetrating hand injury caused by a baiting needle indicated that the incident occurred unexpectedly during routine equipment handling during a competitive feeder fishing event. He reported that he had not anticipated that the injury could result in prolonged hospitalization and subsequent complications, including the need for a secondary scar revision procedure. He emphasized that during competitions actions are performed rapidly and often under time pressure, which may increase the likelihood of accidental injuries. He highlighted the importance of safe storage, proper maintenance, and regular inspection of sharp angling tools to prevent similar events.

The patient with the double-hook finger injury noted that even the preventive practice of carrying metal-cutting pliers did not enable safe hook removal in field conditions due to the hardness and structural characteristics of the hook. He expressed satisfaction with the surgical outcome and full return to activity.

The patient with chronic shoulder and elbow overuse symptoms acknowledged that continued intensive angling during the initial treatment phase contributed to prolonged recovery. He reported that substantial improvement occurred after a marked reduction in angling activity, combined with structured rehabilitation. He subsequently returned to angling with markedly reduced symptoms and greater awareness of load management and preventive conditioning strategies.

## 5. Discussion

### 5.1. Projectile Ocular Injuries Associated with Angling

The present case demonstrates a high-energy projectile mechanism of ocular trauma associated with angling, specifically the sudden snap-back release of a lure from a submerged obstruction. The resulting open-globe injury included a full-thickness corneal laceration, hyphema, and anterior-chamber penetration and required urgent surgical removal of the hook, corneal wound closure, anterior-chamber reconstruction, and evacuation of the hyphema.

Registry data also indicate that fishing-related activities represent a notable proportion of recorded sports-related ocular injuries. In an analysis of the United States Eye Injury Registry, fishing-related activities accounted for 143 of 732 sports-related ocular injuries (19.54%), representing the second most frequently recorded activity after baseball [16].

Fishing-related ocular injuries reported in large emergency-department surveillance datasets are predominantly less severe. Kang et al. [21] identified 141 fishing-related ocular injuries recorded in the U.S. NEISS database between 2014 and 2023, with corneal abrasions representing the most common diagnosis and only a minority of patients requiring hospitalization.

Pak and Kerr [22], analyzing an overlapping NEISS population over a longer period from 2005 to 2024, evaluated 346 fishing-associated ocular injury entries. Most patients (85.2%) were discharged following examination and treatment in the emergency department, whereas 8.1% were admitted. Because both studies were derived from the same surveillance system and included overlapping study periods, their findings should be interpreted as complementary rather than independent. The present case therefore represents the more severe end of the clinical spectrum described in these population-based datasets.

More recently, Rohowetz et al. reported a hospital-based consecutive series of 75 eyes with fishing-related ocular trauma, including 17 open-globe injuries. Fishhook-associated injuries were particularly likely to result in open-globe trauma. Their series also demonstrated variability in perioperative antimicrobial management, including intracameral or intravitreal antibiotics in selected cases and outpatient repair without routine intravenous antibiotics in most documented open-globe injuries, highlighting the individualized nature of management. No new postoperative cases of endophthalmitis were reported [48].

The mechanism observed in our patient is consistent with previous reports showing that hooks, sinkers, and lures released from a tensioned fishing line can become high-velocity projectiles capable of causing severe ocular injury [49].

Purtskhvanidze et al. [45] described open-globe fishhook injuries ranging from isolated anterior-segment wounds to extensive trauma involving the lens, vitreous, retina, and choroid. Similarly, Knox et al. [50] reported penetrating fishhook injuries involving the cornea, anterior chamber, and, in some cases, the lens. In the present patient, visual acuity improved from hand-motion vision to 0.7 at six months. The relatively favorable outcome may reflect the predominantly anterior-segment location of the injury and the absence of lens, retinal, choroidal, or optic-nerve involvement. These observations indicate that prognosis depends not only on the presence of an open-globe injury but also on the anatomical extent of intraocular damage. 

These findings also highlight the potential role of preventive measures. Based on expert guidance and extrapolation from established eye-protection recommendations, the use of impact-resistant wrap-around eyewear with lateral coverage may be considered during lure-based fishing, particularly when casting or attempting to release snagged tackle. Practical precautions may include avoiding forceful traction on a tensioned line directed toward the face or body, keeping bystanders outside the potential recoil trajectory, and using alternative retrieval methods or cutting the line when a snag cannot be released safely. Hoskin et al. additionally emphasized that ordinary prescription spectacles may not provide adequate impact protection and may themselves become a secondary hazard if their lenses shatter following a high-energy impact [49].

### 5.2. Penetrating Hand Injuries Caused by Hooks and Auxiliary Equipment

Cases 2 and 3 illustrate two clinically distinct patterns of penetrating hand injury associated with angling equipment. In Case 2, a baiting needle with a detached handle penetrated deeply into the palmar soft tissues and was not externally visible. Radiography was therefore required to identify the retained approximately 7-cm metallic foreign body, while intraoperative fluoroscopic guidance enabled accurate localization and controlled surgical extraction. An infectious complication occurred during the clinical course, as evidenced by local inflammatory signs, fever, elevated inflammatory markers, and an MSSA-positive wound culture. The infection resolved following antimicrobial treatment, with no subsequent infectious or neurovascular complications. The subsequent clinical course was nevertheless prolonged because palmar scar contracture caused persistent functional limitation, necessitating scar-revision surgery and consideration of an additional corrective procedure. In contrast, Case 3 involved visible transfixion of the finger by a large 10/0 barbed double hook. The entry and exit points and the trajectory of the hook could be assessed clinically, and additional radiographic imaging was not performed. However, the thickness and hardened alloy construction of the hook exceeded the cutting capacity of standard emergency instruments. Mechanical cutting with a grinding device was rejected because of the risk of thermal injury, and controlled surgical extension of the wound tract was required for safe extraction. The patient subsequently reported normal finger function without residual functional limitation.

The predominance of hand and finger involvement in the present cases is consistent with epidemiological evidence. Vajdic et al. [35] reported that the arm and hand were the most frequently affected regions in pediatric fishing-related injuries, while Gil et al. [42] identified the finger and hand as the predominant sites of upper-extremity trauma, with retained foreign bodies representing the most common injury pattern. 

The management of penetrating angling-related injuries depends on the depth and location of the foreign body, the configuration and material properties of the hook or object, and its relationship to tendons, nerves, vessels, joints, and bone. Dudkiewicz et al. [37] emphasized the importance of appropriate imaging and careful planning before extraction, particularly when the object is deeply embedded or its anatomical relationship is uncertain. Standard hook-removal techniques, including retrograde removal, string-yank, needle-cover, and advance-and-cut, may be suitable for selected superficial injuries [51], but were not appropriate in the present cases because of the depth, dimensions, and configuration of the retained objects.

Assessment of distal neurovascular function, possible tendon or joint involvement, contamination, and tetanus status should form part of the initial evaluation. The need for systemic antibiotic therapy should be individualized. While routine prophylaxis is generally not required after uncomplicated superficial hook removal [51], deeper injuries, extensive contamination, retained foreign material, delayed presentation, or involvement of deeper anatomical structures may justify antimicrobial treatment [37]. The injuries described in Cases 2 and 3 therefore should not be generalized to uncomplicated superficial fishhook wounds.

Together, these cases demonstrate that penetrating angling-related hand injuries require individualized assessment rather than a uniform removal strategy. Case 2 was characterized by an occult deeply embedded foreign body and delayed functional impairment due to scar contracture, whereas Case 3 involved a visible but technically difficult double-hook injury, with the patient subsequently reporting normal finger function without residual functional limitation. These differences illustrate how diagnostic and therapeutic decisions may vary according to foreign-body characteristics, anatomical involvement, contamination, and the individual clinical presentation.

### 5.3. Chronic Upper-Limb Overuse Disorders in Anglers

In Case 4, the shoulder and lateral elbow disorders occurred in the context of high annual angling exposure, based on a self-reported retrospective estimate of approximately 200 fishing days per year, together with frequent repetitive casting and rod handling. This exposure estimate is subject to recall and measurement uncertainty. The clinical findings were compatible with cumulative mechanical overload; however, the observational nature of this single case does not allow a causal relationship between angling exposure and the diagnosed musculoskeletal disorders to be established. Although no other regular sporting or strenuous upper-limb activities were reported, prolonged computer-based occupational activity represented a potential additional contributing factor. Age-related or other background musculoskeletal pathology could not be fully excluded. Symptoms improved substantially after a reduction in angling exposure and completion of a structured rehabilitation program, highlighting the potential importance of activity modification and progressive load management.

Survey studies of fly fishers have consistently identified the shoulder and elbow among the most frequently affected upper-extremity regions. Kuhn and Kuhn [6] and Cripps et al. [2] reported associations between upper-extremity symptoms and factors such as casting style, grip configuration, additional line or tackle weight, and high casting exposure. Earlier studies by Berend [29] and McCue et al. [28] similarly suggested that equipment demands and casting technique may influence symptom patterns. However, these studies were based predominantly on self-reported symptoms and therefore demonstrate associations rather than causal relationships with tendon pathology.

Biomechanical data provide a plausible explanation for cumulative upper-limb loading. Allen et al. [52] demonstrated that increasing fly line length alters upper-extremity joint excursions and coordination across the kinetic chain. Although the present patient participated in several angling disciplines rather than fly fishing alone, repeated shoulder rotation, elbow movement, wrist stabilization, and gripping are common across spinning, trolling, and vertical fishing techniques. High-volume participation with limited recovery may therefore contribute to cumulative mechanical loading of the shoulder and elbow, although causality cannot be established from a single case.

Teixeira et al. [30] described a more severe repetitive-stress shoulder injury in an adolescent who developed a partial lesser-tuberosity avulsion after prolonged intensive fly fishing without a single identifiable traumatic event. Although the anatomical lesion differed from the tendinopathy observed in the present patient, the case illustrates that sustained casting exposure may be associated with clinically significant shoulder pathology.

Clinical assessment of anglers with persistent upper-extremity symptoms should therefore consider fishing frequency, session duration, casting volume, tackle load, casting technique, dominant arm, target species, and recovery periods. Management may include temporary reduction in provocative activity, progressive rehabilitation, and gradual return to fishing. Persistent or recurrent symptoms should prompt reassessment and, where clinically indicated, imaging to exclude structural pathology. The available evidence remains largely limited to cross-sectional surveys and isolated case reports; prospective studies using objective exposure measures, biomechanical assessment, and imaging are needed to better define clinically relevant loading patterns and evidence-based preventive strategies.

### 5.4. Prevention and Clinical Implications

Preventive measures should be tailored to the principal mechanism of injury, including projectile, penetrating, and cumulative overuse pathways. The cases presented in this series indicate that injury risk is influenced by the interaction between equipment characteristics, environmental conditions, angling technique, and individual behavior. Accordingly, prevention should combine appropriate protective equipment, safe handling of fishing tackle, awareness of recoil trajectories, regular equipment inspection, and management of repetitive mechanical loading.

Based on expert guidance and extrapolation from established eye-protection recommendations for sports and recreational activities, anglers and nearby observers—particularly children—should consider the use of wrap-around, impact-resistant protective eyewear providing lateral and ultraviolet protection. Polarized lenses may additionally reduce glare and improve visibility below the water surface. Ordinary prescription spectacles should not be regarded as equivalent to protective eyewear specifically designed for impact protection. Fishing has been categorized by the American Academy of Ophthalmology and the American Academy of Pediatrics as an activity associated with a moderate risk of ocular injury, supporting consideration of appropriate eye protection [53].

Additional preventive measures proposed in the literature include barbless or retractable hooks, hook guards, magnetic hook-retrieval tools, and equipment systems designed to limit uncontrolled line recoil. Practical instruction may emphasize safe casting, adequate spacing between participants, awareness of nearby observers, and the hazards created by hooks, lures, and weights released from a tensioned line. Educational videos, organized safety programs, and warning signage at frequently used fishing locations may further improve awareness [21,40,49]. These measures should be regarded as practical safety proposals and expert opinion rather than evidence-based interventions, as barbless or retractable hooks, hook guards, and similar devices have not been shown in interventional studies to reduce the incidence of angling-related injuries.

Particular caution is required when releasing a lure or hook snagged on an underwater or shoreline obstruction. Anglers may be advised to avoid pulling a highly tensioned line directly toward the face or body and should ensure that no other person is positioned within the potential recoil trajectory. Retrieval should initially be attempted by changing the direction of traction or using a dedicated lure-retrieval device. When the snag cannot be released safely, cutting the line may be preferable to repeated forceful pulling. These precautions directly address the snap-back mechanism observed in the present ocular case and in previously reported injuries in which hooks caught in trees or bushes recoiled toward the eye after the line was tensioned [45,49,50].

Practical preventive measures may include storing hooks, baiting needles, and other sharp accessories in closed, clearly organized compartments. Regular inspection of equipment may help identify loose or damaged handles that may leave sharp metallic components exposed or concealed among other accessories. Covering hook points during handling and transport may be considered. Anglers using large hooks manufactured from hardened alloys may consider carrying cutting tools capable of severing the specific hook type, because standard pliers may be ineffective against thick treble or double hooks. Nevertheless, field removal should not be attempted when a hook is deeply embedded, transfixes the finger or hand, involves anatomically important structures, or cannot be removed without substantial tissue manipulation [40,51].

For highly active anglers, preventive strategies may also address cumulative mechanical loading. Based on extrapolation from general sports-medicine and overuse-injury prevention principles, casting and retrieval volume may be increased gradually, with recovery periods between sessions and variation in fishing techniques. The onset of shoulder, elbow, wrist, or forearm pain may warrant early activity modification rather than continued fishing despite symptoms. Preventive conditioning may include rotator-cuff and scapular-stabilizer strengthening, forearm flexor and extensor endurance training, grip conditioning, trunk control, mobility exercises, and attention to efficient casting biomechanics. Structured warm-up and adequate recovery between intensive sessions may also be reasonable preventive strategies; however, neither warm-up nor conditioning programmes have been demonstrated in interventional studies to reduce angling-related injury incidence [2,6,10,28].

From a clinical perspective, fishing-related mechanisms should be actively considered when evaluating ocular trauma, penetrating hand injuries, or otherwise unexplained upper-limb pain in recreational and competitive anglers. In ocular trauma, clinicians should determine whether the injury is confined to the eyelid or represents an open-globe injury, because treatment complexity and visual prognosis depend largely on the anatomical depth of penetration and the structures involved. An embedded hook should be stabilized rather than removed blindly, and urgent ophthalmologic assessment is required whenever globe penetration is suspected. The choice of removal technique should take into account hook size, curvature, material, number of points, barb configuration, visibility of the tip, and involvement of the cornea, sclera, iris, lens, or posterior segment [40,45,50].

For penetrating extremity injuries, clinical assessment should include tendon function, joint involvement, distal circulation, sensory and motor nerve function, and the possibility of a retained foreign body. Radiography, ultrasonography, or intraoperative imaging may be required when the object is not fully visible or its trajectory cannot be reliably established. Management should also consider contamination by freshwater, fish mucus, bait, soil, or other organic material, together with appropriate wound irrigation, tetanus prophylaxis, antimicrobial treatment when clinically indicated, and follow-up for infection, scar contracture, and functional impairment [37,47].

In patients with overuse disorders, clinical management should extend beyond symptomatic pharmacological treatment. Assessment of fishing frequency, session duration, casting volume, retrieval technique, weight, recovery periods, and other occupational or sporting exposures may help identify the principal mechanical contributors. Rehabilitation should be combined with temporary load reduction and a gradual return to angling. Together, these measures provide a mechanism-based approach linking prevention with early recognition, appropriate specialist referral, individualized treatment, and functional recovery [2,6,10].

### 5.5. Strengths and Limitations

This case series provides clinically detailed examples of angling-related injuries across different fishing disciplines and offers individual clinical context that complements evidence derived from survey-based and registry studies. Nevertheless, the descriptive design limits generalizability, and incidence rates cannot be inferred from these data.

Case identification was non-systematic and based on purposive, multimodal recruitment, including questionnaire-based data collection, dedicated online angling forums, angling events, and direct contact through the Gdańsk and Bydgoszcz Districts of the Polish Angling Association (PZW). Recruitment was conducted within a single country and was geographically centered on two regional districts, which further limits the generalizability of the findings to other angling populations and healthcare settings. The presented cases were selected to illustrate clinically relevant and distinct injury mechanisms rather than to reflect their frequency or distribution in the broader angling population. Although participant flow within the questionnaire arm could be reconstructed, no prospective screening log was maintained across the other recruitment channels; therefore, the exact number of potentially eligible individuals who did not proceed to formal inclusion could not be reliably determined.

The purposive and multimodal recruitment strategy may have preferentially captured more severe, unusual, or memorable injuries and therefore introduced selection and reporting bias. Patient-reported injury mechanisms and exposure histories may also be subject to recall bias. In addition, the small number and clinical heterogeneity of the cases and incomplete standardization of diagnostic work-up and follow-up restrict the ability to draw definitive conclusions regarding risk factors, comparative treatment effectiveness, or causal relationships. 

Retrospectively collected patient perspectives may be affected by recall and social-desirability bias.

Validated patient-reported outcome measures were available only at the late investigator-conducted follow-up for Cases 2 and 4, without corresponding baseline or serial measurements, which limits longitudinal interpretation of functional outcomes. No validated PROMs were available for Cases 1 and 3.

An additional limitation is that clinical verification relied on medical documentation voluntarily supplied by participants rather than on direct access to complete institutional medical records. Imaging findings were assessed from the available written radiological or ultrasonographic reports rather than through independent review of the original images. Consequently, some clinical information may have been incomplete, and the possibility of diagnostic misclassification cannot be entirely excluded.

Despite these limitations, the cases provide practical clinical examples of distinct angling-related injury mechanisms and complement population-level surveillance data by illustrating individual clinical presentations and management courses.

## 6. Conclusions

Recreational and sport angling may be associated with a range of acute and chronic injuries, including ocular trauma, penetrating hand injuries, and upper-limb overuse disorders. The four purposively selected cases presented in this series illustrate clinically distinct injury mechanisms and management challenges but cannot establish the frequency of these injuries or the effectiveness of preventive strategies.

From a clinical perspective, awareness of angling-related mechanisms may assist in the assessment of ocular, hand, and upper-limb conditions in recreational and competitive anglers. The present cases also highlight the potential value of patient education regarding injury mechanisms and safe equipment handling. Preventive measures discussed in this report should be regarded as clinical considerations and hypotheses for further study rather than as interventions of established effectiveness.

Larger, systematically recruited prospective studies are needed to better characterize the epidemiology, risk factors, clinical outcomes, and potential preventive strategies associated with angling-related injuries.

## Figures and Tables

**Table 1:** Clinical timelines of the cases.

Case	Date	Clinical Course, Management, and Outcome
Case 1—Ocular trauma	12 May 2024	High-velocity lure impact to the right eye causing an open-globe injury with corneal laceration, hyphema, and severe visual impairment; CT confirmed hook penetration into the anterior chamber.
	12 May 2024	Urgent hook removal, corneal suturing, anterior-chamber reconstruction, and hyphema evacuation; antimicrobial and anti-inflammatory treatment initiated.
	13 May 2024	IOP measured at 14 mmHg; postoperative ophthalmological assessment performed.
	20 May 2024	Close ophthalmological follow-up. IOP 15 mmHg. BCVA improved to 0.4.
	28 May 2024	2-week follow-up of corneal healing, sutures, and potential complications; IOP 15 mmHg, BCVA 0.4.
	24 June 2024	6-week follow-up of corneal healing, sutures, and potential complications; IOP 14 mmHg, BCVA 0.4.
	12 August 2024	3-month follow-up of corneal healing, sutures, and potential complications; IOP 14 mmHg, BCVA 0.5.
	19 November 2024	At 6 months, BCVA improved to 0.7, IOP 16 mmHg, mild irregular astigmatism persisted, with no infectious or posterior-segment complications.
	15 November 2025	Participant identified and recruited into the study; written informed consent obtained.
	17 November 2025	Participant formally enrolled in the study.
	20 November 2025	Investigator-conducted interview performed to retrospectively reconstruct the circumstances and clinical course of the injury.
Case 2—Penetrating palmar hand injury	15 June 2023	Penetrating injury of the right palm caused by a retained baiting needle; radiography demonstrated an approximately 7-cm metallic foreign body.
	15 June 2023	Surgical extraction under fluoroscopic guidance; tetanus prophylaxis.
	16 June 2023	Antimicrobial treatment initiated.
	15–20 June 2023	Six-day hospitalization for an infectious complication with fever, leukocytosis, elevated CRP, and local inflammatory signs; wound culture yielded MSSA susceptible to amoxicillin–clavulanate. IV therapy was followed by oral antimicrobial treatment.
	14 September 2023	3-month follow-up during structured hand rehabilitation including range-of-motion exercises, tendon-gliding, scar mobilization, progressive grip strengthening, and functional training.
	16 November 2023	After completion of rehabilitation, hand mobility, grip strength, and pain improved, although residual palmar scar contracture persisted.
	29 June 2024	Scar revision surgery performed because of persistent post-traumatic palmar scar contracture and functional limitation.
	17 July–11 September 2024	Further 8-week structured hand rehabilitation after scar revision
	8 July 2025	Late follow-up showing further improvement but persistent residual scar contracture and functional limitation; an additional corrective procedure was being considered.
	4 January 2026	Participant identified and recruited through direct in-person contact via the Polish Angling Association; written informed consent obtained.
	6 January 2026	Participant formally enrolled in the study.
	15 April 2026	QuickDASH: 22.7/100 at investigator-conducted follow-up
Case 3—Double-hook finger injury	10 August 2024	Large 10/0 barbed double hook penetrated the soft tissues of the left ring finger. No clinical evidence of neurovascular or tendon injury was identified.
	10 August 2024	Controlled surgical removal through limited extension of the wound tract, followed by irrigation. Oral ciprofloxacin and tetanus prophylaxis were administered. No cultures or laboratory investigations were performed.
	22 August 2024	Suture removal after satisfactory wound healing.
	10 September 2024	One-month follow-up showed uncomplicated wound healing with preserved tendon integrity and intact sensory, motor, and vascular function. The patient resumed angling and reported no clinically relevant functional impairment.
	09 April 2026	Participant identified through the study questionnaire and recruited.
	10 April 2026	Written informed consent obtained.
	11 April 2026	Medical documentation was voluntarily provided; later, a photograph obtained approximately 18 months after the procedure documented the appearance of the healed finger.
	13 April 2026	Participant formally enrolled in the study.
	15 April 2026	Investigator-conducted follow-up: the patient reported no pain, sensory disturbance, motor impairment, or residual functional limitation.
Case 4—Rotator-cuff tendinopathy and lateral epicondylitis	Approximately December 2019	Gradual development of right shoulder and right lateral elbow symptoms in the context of high angling exposure and repetitive casting and rod handling.
	5 May 2020	Shoulder and elbow pain rated 7/10 on the NRS, with pain-limited shoulder abduction/external rotation and pain on resisted wrist extension. Ultrasonography showed supraspinatus tendinopathy without full-thickness tear; lateral epicondylitis was diagnosed clinically.
	5 May 2020	Conservative treatment initiated, including diclofenac 50 mg twice daily for 10 days, reduction in provocative angling activity, and a 12-week structured physiotherapy program.
	3 August 2020	Pain decreased from 7/10 to 2/10 on the NRS, with substantial functional improvement and return to angling. No post-treatment goniometric ROM, grip-strength, or validated functional outcome measures were available.
	20 January 2026	Participant identified and recruited.
	10 February 2026	Written informed consent obtained.
	14 April 2026	Participant formally enrolled in the study.
	15 April 2026	Investigator-conducted retrospective follow-up interview; intermittent symptom recurrence 1–2 times/year. SPADI 10.8/100; PRTEE 0/100.

**Table 2:** Demographic and angling characteristics of the four patients included in the case series.

Case	Clinical Presentation	Age (Years)	Sex	Angling Experience (Years)	Angling Profile
1	Ocular trauma	34	Male	20	Active angling; spinning
2	Penetrating palmar hand injury	39	Male	25	Feeder fishing; competitive angling
3	Penetrating finger injury	40	Male	25	Active angling; spinning; catfish fishing; competitive angling
4	Rotator cuff tendinopathy and lateral epicondylitis	40	Male	30	Active angling; spinning

**Table 3:** Summary of fishing-related injury cases.

Case	Injury Type	Mechanism	Location	Side	Management	Outcome
1	Ocular trauma	High-velocity lure (snap-back)	Eye	Right	Urgent surgical repair (corneal suturing)	Substantial but incomplete visual recovery (BCVA 0.7 at 6 months)
2	Penetrating injury	Baiting needle	Hand (palmar region)	Right	Surgical removal + antibiotics + rehabilitation	Persistent scar-related functional limitation; further corrective procedure was being considered
3	Penetrating injury	Large double hook	Ring finger	Left hand	Surgical removal	Patient-reported complete recovery without residual functional limitation
4	Overuse disorder	Repetitive casting	Shoulder & elbow	Right	Rehabilitation + load reduction	Substantial clinical and functional improvement, with intermittent symptom recurrence

## Data Availability

The data supporting the findings of this case series are contained within the article. Additional anonymized data may be made available by the corresponding author upon reasonable request, subject to applicable ethical, institutional, and patient confidentiality restrictions. The data are not publicly available due to the small number of cases and the potential risk of indirect patient identification.

## Supplementary Materials

The following supporting information can be downloaded at: https://www.mdpi.com/article/10.3390/healthcare14182977/s1. CARE Checklist of information to include when writing a case report.
