# Supplementary material for: Injuries Associated with Recreational and Sport Angling: A Case Series of Mechanisms, Diagnosis, Treatment, and Rehabilitation
Source: Healthcare (Basel). 2026 Sep 11;14(18):2977. doi: 10.3390/healthcare14182977 (PMC13606276; doi:10.3390/healthcare14182977)
Supplement: Supplementary file 1 [file healthcare-14-02977-s001.zip › healthcare-4485874-supplementary.pdf]

CARE
case report guidelines

CARE Checklist of information to include when writing a case report

(cc) BY-NC-ND

Topic	Item	Checklist item description	Reported on Line
Title	1	The diagnosis or intervention of primary focus followed by the words "case report"	Title page (case series)
Key Words	2	2 to 5 key words that identify diagnoses or interventions in this case report, including "case report"	Keywords (case series)
Abstract (no references)	3a	Introduction: What is unique about this case and what does it add to the scientific literature?	Abstract - Background, p. 1
	3b	Main symptoms and/or important clinical findings	Abstract - Results, p. 1
	3c	The main diagnoses, therapeutic interventions, and outcomes	Abstract - Results, p. 1
	3d	Conclusion—What is the main "take-away" lesson(s) from this case?	Abstract - Conclusions, p. 1
Introduction	4	One or two paragraphs summarizing why this case is unique (**may include references**)	Section 1 - Background, pp. 1-2
Patient Information	5a	De-identified patient specific information.	Secs. 3.1-3.4; Table 2, pp. 6-16
	5b	Primary concerns and symptoms of the patient.	Secs. 3.1-3.4, pp. 6-14
	5c	Medical, family, and psycho-social history including relevant genetic information	Secs. 3.1-3.4, pp. 6-14 (as available)
	5d	Relevant past interventions with outcomes	Secs. 3.1-3.4, pp. 6-14 (as applicable)
Clinical Findings	6	Describe significant physical examination (PE) and important clinical findings.	Secs. 3.1-3.4, pp. 6-14
Timeline	7	Historical and current information from this episode of care organized as a timeline	Table 1, pp. 14-16
Diagnostic Assessment	8a	Diagnostic testing (such as PE, laboratory testing, imaging, surveys).	Secs. 3.1-3.4; Table 1, pp. 6-16
	8b	Diagnostic challenges (such as access to testing, financial, or cultural)	Secs. 3.1 & 3.3, pp. 6-12 (as applicable)
	8c	Diagnosis (including other diagnoses considered)	Secs. 3.1-3.4, pp. 6-14
	8d	Prognosis (such as staging in oncology) where applicable	N/A; case-specific follow-up in Secs. 3.1-3.4
Therapeutic Intervention	9a	Types of therapeutic intervention (such as pharmacologic, surgical, preventive, self-care)	Secs. 3.1-3.4, pp. 7-14
	9b	Administration of therapeutic intervention (such as dosage, strength, duration)	Secs. 3.1-3.4, pp. 7-14
	9c	Changes in therapeutic intervention (with rationale)	Secs. 3.1-3.4, where applicable
Follow-up and Outcomes	10a	Clinician and patient-assessed outcomes (if available)	Secs. 3.1-3.4; Tables 1 & 3, pp. 7-16
	10b	Important follow-up diagnostic and other test results	Secs. 3.1-3.4; Table 1, pp. 7-16
	10c	Intervention adherence and tolerability (How was this assessed?)	Sec. 3.4, p. 13; others N/A
	10d	Adverse and unanticipated events	Secs. 3.1-3.4; Table 1, as applicable
Discussion	11a	A scientific discussion of the strengths AND limitations associated with this case report	Sec. 5.5 - Strengths/limitations, p. 23
	11b	Discussion of the relevant medical literature **with references**.	Secs. 5.1-5.4 - Discussion, pp. 17-23
	11c	The scientific rationale for any conclusions (including assessment of possible causes)	Secs. 5.1-5.4; Sec. 6, pp. 17-24
	11d	The primary "take-away" lessons of this case report (without references) in a one paragraph conclusion	Sec. 6 - Conclusions, p. 24
Patient Perspective	12	The patient should share their perspective in one to two paragraphs on the treatment(s) they received	Sec. 4 - Patient Perspective, p. 17
Informed Consent	13	Did the patient give informed consent? Please provide if requested	Yes ☒ No ☐

Consent location: Methods pp. 3-4; Informed Consent Statement p. 24
